# Perceived needs and satisfaction with care in people with multiple sclerosis: A two-year prospective study

**DOI:** 10.1186/1471-2377-8-36

**Published:** 2008-09-29

**Authors:** Charlotte Ytterberg, Sverker Johansson, Kristina Gottberg, Lotta Widén Holmqvist, Lena von Koch

**Affiliations:** 1 Department of Clinical Neuroscience, and Department of Neurobiology, Care Sciences and Society, Karolinska Institutet, Stockholm, Sweden; 2 Department of Neurobiology, Care Sciences and Society, Karolinska Institutet, Stockholm, Sweden

## Abstract

**Background:**

Considering the costs of multiple sclerosis (MS), it is crucial that the health-related services supplied are in accordance with needs as they are perceived by people with MS (PwMS). Satisfaction with care is related to quality of care and can provide health care providers with the means for improvement. The aim was to explore the perceived needs and satisfaction with care amongst PwMS over a two-year period, also taking sex and disease severity into consideration.

**Methods:**

The sample consisted of 219 outpatients at a MS specialist clinic. Data on perceived needs and satisfaction with care were collected every six months using a questionnaire which included various dimensions of care. The data was analysed for the whole sample and on an individual level, as well as in subgroups with regard to sex and disease severity.

**Results:**

There were no statistically significant variations in the proportion of PwMS with perceived needs concerning different health-related services during the study period. However, individual variations were found with regard to both perceived needs and satisfaction with care. Few PwMS perceived a continuous need for a specific service. However, the majority perceived a need for rehabilitation, assistive devices, transportation service for the disabled, psychosocial support/counselling and information on social insurance/vocational rehabilitation at least sometimes. Severe MS was associated with a greater perceived need for almost all the services studied and women experienced a need for psychosocial support/counselling to a greater extent than men. In relation to the different categories of health care staff, PwMS were most satisfied with nurses with regard to all dimensions of care. They were least satisfied with the availability of psychosocial support/counselling; and information about social insurance/vocational rehabilitation.

**Conclusion:**

Despite the large proportion of individuals with mild disease severity in our sample, a considerable number of needs were identified of which many, on an individual level, varied over time. Key services demanded by PwMS were identified. Also the level of satisfaction with care varied and areas with a potential for improvement were identified such as the availability of rehabilitation services including an increase in the supply of psychosocial support and counselling.

## Background

Multiple sclerosis (MS) is a chronic, neurological disease, the onset of which usually occurs between the ages of 20–40 years. The disease results in a wide range of symptoms, but does not alter the life span substantially [1]. Consequently the majority of people with MS (PwMS) live with the disease for most of their lives and over a period of many years experience various needs for health-related services.

A recent study revealed that the great majority of PwMS in Stockholm make parallel use of hospital specialist care and primary care and that neurology care contacts constitute only 20% of all outpatient care [2]. The total cost of MS in Sweden is estimated at 600 million euro per year, of which direct costs account for 68% [3]. Considering the extensive costs of MS in terms of public spending, it is crucial that the health-related services supplied are effective and in accordance with needs as perceived by the PwMS. It has been shown that patients' and doctors' perceptions of disability in MS differ [4] and disabled people and their nominated key professionals have been found to perceive needs for health-related services differently [5]. It is therefore important to identify the needs that are perceived by the PwMS and to investigate their satisfaction with care.

Satisfaction with care is a complicated, multidimensional concept that includes both affective and cognitive components [6]. It is related to quality of care [7,8] and can provide health care providers with the means for improvement. A number of studies have revealed that PwMS are dissatisfied with several areas of care e.g., rehabilitation, psychosocial counselling, adaptations, coordination between services and advice concerning MS-related matters [2,9-14]. However, few studies [9] have explored the variation in perceived needs and satisfaction with care over time in PwMS and potential differences between subgroups (e.g., with regard to sex and disease severity) have been sparsely studied [9,11,14]. Such knowledge is important for the planning of health-related services for PwMS to be able to meet perceived needs and optimise patient satisfaction.

The International Classification of Functioning, Disability and Health (ICF) aims to provide a scientific basis for the study of health conditions and is also useful for the study of health care systems, in terms of outcomes and the cost-effectiveness of services, the assessment of needs and consumer satisfaction [15]. The ICF has two parts: 1) Functioning, including body functions/body structures, and activities and participation, 2) Contextual Factors, including environmental and personal factors, which determine the level and extent of the individual's functioning. Environmental factors e.g., health-related services, make up the physical, social and attitudinal environment; by not providing adequate health-related services, society may create environmental barriers instead of providing facilitators [15]. Satisfaction with care is not classified within the ICF framework but may be regarded as an environmental factor. For studies of satisfaction with care Ware et al. have proposed a theoretical framework which includes eight dimensions that should be assessed [16]. This taxonomy has previously been applied in several studies of people with neurological disorders [2,17,18].

The aim of the present study was to explore perceived needs in relation to different health-related services, also taking sex and disease severity into consideration, and to explore PwMS's satisfaction with care over a two-year period.

## Methods

### Participants and procedures

The study was carried out in the context of a prospective, observational study of functioning and perceived health amongst PwMS [19]. Those eligible were all PwMS diagnosed according to the Poser criteria [20], who, during the period from February 1, 2002 to June 12, 2002, were scheduled for an outpatient appointment with one of two senior neurologists at the MS Centre of the Department of Neurology, Karolinska University Hospital, Huddinge, in Stockholm, Sweden. The PwMS received written and oral information about the study and were included after having given their informed consent. The PwMS were followed up every six months for two years, in the first place in conjunction with their regular visits to the senior neurologist, otherwise an appointment was made for data-collection on another day. At the time of inclusion and at 6, 12, 18 and 24 months, the PwMS met an investigator, one of five research physiotherapists, primarily the same investigator and at the same time of day on all occasions.

The neurologist responsible determined disease course and assessed disease severity by means of the Expanded Disability Status Scale (EDSS) [21]. EDSS-scores were categorized according to the Swedish MS-register as EDSS normal (0), EDSS mild (1.0–3.5), EDSS moderate (4.0–5.5) and EDSS severe (6.0–9.5) [22]. The remaining data was collected by the research physiotherapists. Demographic data and clinical characteristics were collected by means of interviews and from the medical records.

Data regarding perceived needs and satisfaction with care was collected using a questionnaire that has previously been used in studies of people with stroke [18], Guillain-Barré syndrome [17] and MS [2]. The questionnaire was based on Ware's taxonomy [16], and included the dimensions of art of care (engagement/sympathy, kind treatment), accessibility, technical quality of care, finances, availability, continuity and efficacy/outcomes of care. The questionnaire has been found to be feasible including good face validity. In addition to the dimensions suggested by Ware, one item dealing with participation in planning care was included in a previous study of people with stroke [18], since patient participation is included in Sweden's Health and Medical Services Act. Furthermore, as a result of a pilot study exploring the feasibility of the instrument among PwMS [23], items dealing with diagnosis-related information and information about the disease were included in a previous population-based study of people with multiple sclerosis in Stockholm County [2]. The questionnaire consisted of 22 statements, which the PwMS were asked to agree or disagree with on a 5-graded Likert scale. The alternative "not applicable" was also presented. Data on all 22 items included in the questionnaire was collected on inclusion, whereas data regarding the ten items related to availability was collected on inclusion and at 6, 12, 18 and 24 months. The items regarding availability consisted of ten statements concerning different health-related services e.g., physiotherapy, assistive devices and transportation services for the disabled. For each service, the PwMS were asked to choose between the alternatives "need" or "no need" and, for each service need indicated, they were asked to rate agreement on a 5-graded Likert scale e.g., "I have received all the physiotherapy that I need".

The study was approved by the ethical committee of Karolinska Institutet in Stockholm, Sweden.

### Statistical analysis

Descriptive statistics were used to present perceived need and satisfaction with care. Satisfaction with care was dichotomised into satisfied (1–2 on the Likert scale) or not satisfied (3–5 on the Likert scale). The Cohran Q test was performed for analyses of the changes in the proportion of PwMS with perceived needs during the study period with regard to different health-related services for the whole sample and for the subgroups, with regard to sex and disease severity. Chi square statistics were used for the analyses of differences in sex and disease severity between PwMS with perceived needs for different health-related services and those with no perceived need, and between PwMS who were satisfied with availability and those who were not. A p value ≤ 0.05 was considered statistically significant. The statistical analyses were performed in Statistica 7.

## Results

Of 255 eligible PwMS, 36 declined and 219 were included in the study. During the study period seven PwMS died and 12 withdrew, leaving 200 who were followed for two years. Demographic and clinical characteristics on inclusion are summarised in Table 1.

**Table 1:** Demographic and clinical characteristics of PwMS on inclusion

	n (%)
People with MS	219
Women	149 (68)
Age, years	47 (12, 20–75) *
Living with a partner	152 (69)
Working full or part time †	117 (58)
Disease course:	
Relapsing remitting	127 (58)
Secondary progressive	83 (38)
Primary progressive	9 (4)
Disease severity:	
EDSS normal, 0	1 (0.5)
EDSS mild, 1–3.5	129 (59)
EDSS moderate, 4–5.5	37 (17)
EDSS severe, 6–9.5	52 (23.5)
Time lapse since diagnosis, years	14 (10, 0–44) *
Immunomodulatory treatment	182 (83)

* mean (SD, range)

† Working; used only for PwMS < 65 years of age, the customary age for retirement in Sweden

Perceived need and PwMS's satisfaction with care on inclusion are presented in Table 2. All the PwMS preferred an early diagnosis but only around half were satisfied with the circumstances under which the diagnosis was delivered. Most PwMS were satisfied in areas concerning information about the disease, art of care (engagement/sympathy, kind treatment) and accessibility, although fewer were satisfied with their contacts with psychologists and access to physicians. As far as the professional health care staff were concerned, these PwMS were most satisfied with nurses when it came to all dimensions of care. For the majority of the participants, all the expertise needed had been available, but most of those who perceived a need for information regarding social insurance/vocational rehabilitation and half of those who perceived a need for psychosocial support/counselling and rehabilitation periods had not received these services to the extent that they perceived necessary.

**Table 2:** Perceived need and satisfaction with care of PwMS (n = 219) on inclusion

Dimensions	Applicable/Perceived need, %	Satisfied, %*
*Diagnosis-related information*		
Want early diagnosis	100	84†
Content with situation in which the diagnosis was received	100	57
*Information regarding the disease*		
Physicians	100	81
Nurses	86	93
Occupational therapists	37	70
Physiotherapists	52	72
Welfare officers	37	67
Psychologists	13	54
Others	28	77
*Art of care*		
Engagement/sympathy from staff		
Physicians	100	82
Nurses	89	88
Occupational therapists	34	75
Physiotherapists	50	85
Welfare officers	35	76
Psychologists	13	56
Others	26	79
Kind treatment		
Physicians	100	90
Nurses	90	96
Occupational therapists	36	86
Physiotherapists	53	88
Welfare officers	35	85
Psychologists	13	67
Others	30	81
*Accessibility*		
Physicians	84	65
Nurses	85	81
Occupational therapists	28	78
Physiotherapists	44	77
Welfare officers	26	71
Psychologists	9	55
Others	23	77
*Technical quality of care*		
Contact with all expertise needed	98	82
*Finances*	98	25
*Availability*		
Physiotherapy	61	65
Occupational therapy	41	66
Rehabilitation periods	42	58
Assistive devices	45	80
Workplace adaptation	28	70
Home adaptation	34	79
Transportation service for the disabled	42	89
Home help/Personal assistants	21	66
Psychosocial support/counselling	38	50
Information on social insurance/vocational rehabilitation	40	29
*Continuity*		
Meeting the same staff	98	88
*Participation in planning care*		
Want to participate in planning care	95	85†
Have participated in planning care	79	63
*Efficacy/outcomes of care*		
Hospital inpatient care	48	80
Hospital outpatient care	97	89
Primary care	52	67
Rehabilitation periods	32	78

* Among those who perceived need

† Agree

Despite the fact that most of the PwMS in the sample had mild EDSS, a high proportion perceived a need for physiotherapy (61%); occupational therapy (41%); and rehabilitation periods (a defined time-period of more intense rehabilitation at special rehabilitation units or in day care) (42%). However, more than one third of those who perceived a need were not satisfied with these areas of rehabilitation. The costs of care were considered burdensome by 75%. The vast majority wanted to participate in planning their care but only 63% of those who perceived a need to participate were satisfied. Regarding continuity and efficacy/outcomes of care, the majority were satisfied.

During the study period there were no statistically significant variations over time in the proportion of PwMS with perceived needs concerning different health-related services; either in the whole sample or with regard to sex. In those with severe EDSS, a statistically significant variation was found concerning occupational therapy (p = 0.007). Table 3 presents those PwMS who, in the course of the study a) perceived needs of the different health-related services at all times (inclusion, 6, 12, 18 and 24 months), sometimes or never, and b) of those who perceived a need, the proportion of PwMS who were satisfied at all times, sometimes or never. Individual variations in perceived needs as well as in satisfaction with care were found regarding most health-related services. Few PwMS perceived a persistent need for a specific health-related service during the period of the study. However, the majority perceived a need for rehabilitation (physiotherapy – 85%, occupational therapy – 62%, rehabilitation periods – 60%), assistive devices (69%), transportation service for the disabled (62%), psychosocial support/counselling (60%) and information on social insurance/vocational rehabilitation (57%) at all times or sometimes. Of those who perceived a need at all times or sometimes, these PwMS were most satisfied with the availability of assistive devices; workplace adaptation; home adaptation; and transportation service for the disabled. PwMS were least satisfied with the availability of psychosocial support/counselling; and information on social insurance/vocational rehabilitation. Among those who perceived a persistent need, the transportation service for the disabled was the only item where more PwMS were satisfied at all times than sometimes.

**Table 3:** Number of PwMS who during the study a) perceived needs of the different health-related services at all times (inclusion, 6, 12, 18 and 24 months), sometimes or never, and b) of those who perceived a need, the proportion of PwMS who were satisfied at all times, sometimes or never

	Need, n (%)	Satisfied, %		
		All times	Sometimes	Never
*Physiotherapy*				
All times	53 (26)	40	60	0
Sometimes	118 (59)	42	37	20
Never	29 (14)			
*Occupational therapy*				
All times	11 (6)	54	45	0
Sometimes	114 (57)	48	31	21
Never	75 (37)			
*Rehabilitation periods*				
All times	11 (6)	0	91	9
Sometimes	110 (55)	40	29	31
Never	79 (40)			
*Assistive devices*				
All times	10 (5)	50	50	0
Sometimes	129 (64)	62	26	12
Never	61 (30)			
*Workplace adaptation*				
All times	1 (0)	-	-	-
Sometimes	90 (45)	56	13	31
Never	109 (54)			
*Home adaptation*				
All times	0 (0)	-	-	-
Sometimes	104 (52)	58	18	24
Never	96 (48)			
*Transportation service for the disabled*	40 (20)	68	32	0
All times	84 (42)	76	14	10
Sometimes	76 (38)			
Never				
*Home help/Personal assistants*				
All times	10 (5)	30	70	0
Sometimes	73 (36)	46	23	30
Never	117 (58)			
*Psychosocial support/Counselling*				
All times	4 (2)	0	75	25
Sometimes	116 (58)	46	16	38
Never	80 (40)			
*Information on social insurance/vocational rehabilitation*				
All times	1 (0)	0	100	0
Sometimes	113 (56)	24	14	62
Never	86 (43)			

Women experienced a need for psychosocial support/counselling to a greater extent than men (p = 0.04). Between PwMS with mild and severe EDSS there were statistically significant differences with more people among those with severe EDSS perceiving needs. PwMS with moderate EDSS did not differ in perceived needs from those with mild or those with severe EDSS. A severe state of MS was associated with a greater perceived need and a mild state of MS was associated with a smaller perceived need for the availability of almost all the health-related services studied: physiotherapy (p = 0.004), occupational therapy (p = 0.01), rehabilitation periods (p = 0.008), assistive devices (p = 0.002), home adaptation (p = 0.04), transportation service for the disabled (p = 0.002) and home help/personal assistants (p < 0.001). However, there were no associations between disease severity and perceived need for workplace adaptation, psychosocial support/counselling and information on social insurance/vocational rehabilitation; nor was there any association between satisfaction with care and sex or disease severity.

## Discussion

This study explored the perceived needs and satisfaction with care over a period of two years at a MS specialist outpatient clinic, taking sex and disease severity into consideration. Individual variations were found with regard both to perceived needs and satisfaction with care. However, the majority of the participants perceived a need for rehabilitation, assistive devices, transportation service for the disabled, psychosocial support/counselling and information on social insurance/vocational rehabilitation at all times or sometimes. A severe state of MS was associated with a greater perceived need for almost all the health-related services studied and women experienced a need for psychosocial support/counselling to a greater extent than men.

The majority preferred an early diagnosis, a result which tallies with previous studies [2,24]. However, only around half were satisfied with the circumstances surrounding the presentation of the diagnosis. This finding might reflect the fact that some PwMS in the sample received their diagnosis many years ago at a time when the diagnostic process was slower than today. The stress associated with receiving a MS diagnosis [25] may also have a negative impact on the degree of satisfaction. Nevertheless, there is probably some potential for improvement with regard to the diagnostic situation and this possibility should be further explored.

Regarding contacts with the different health care providers, the PwMS were least satisfied with the psychologists with regard to such matters as information about the disease, art of care (engagement/sympathy, kind treatment) and accessibility. One possible explanation can be that contacts with psychologists will most likely include an assessment of cognitive functioning, which, for the individual, may be associated with feeling ill-at-ease [26].

Despite the fact that the PwMS met a neurologist every six months, as many as 35% of them were not satisfied with the physicians' accessibility; this is a result in line with a previous study [2]. This finding might reflect a need for more frequent visits or telephone contacts but may also illustrate the fact that physicians are the gate keepers to many health-related services. Previous studies have recognised the need for more effective care coordination [9,27]. Future studies should identify the issues underlying the call for increased access to physicians and explore how these needs could best be met e.g., by involving a multi-professional team or a designated care coordinator [28].

As far as the professional health care staff is concerned, the PwMS were most satisfied with nurses with regard to all the dimensions studied. A plausible reason for this result may be that the majority of these PwMS received immunomodulatory treatment, entailing repeated contacts with nurses for instructions and assistance with the injections. This frequent contact may lead to the nurses becoming care coordinators.

Regardless of disease severity, the majority of those who indicated a need for information regarding social insurance/vocational rehabilitation had not received this service to the extent that they perceived necessary. In a country like Sweden, with a public social insurance system, an improved information service regarding these issues as well as access to vocational rehabilitation services that respond to individual needs [29] might be a facilitating environmental factor which could enable PwMS to work and might facilitate participation.

Only half of those who perceived a need for psychosocial support/counselling reported that they had received this service to the extent that they perceived necessary; this is in line with results from a previous study [2] and possibly reflects the fact that this kind of service is not only in poor supply within the public social insurance system in Sweden but is also mainly provided by private caregivers, meaning high costs for the individual.

The cost of rehabilitation is relatively small. Nevertheless, a recent study of the costs of MS in Sweden revealed that the expenditure on rehabilitation was reduced from 8.6% in 1998 to 7.2% in 2005 [3]. Considering that large proportions reported a need for physiotherapy, occupational therapy, and periods of rehabilitation and that more than one third were not satisfied with the care situation, the rationale for the reduction in rehabilitation costs can be questioned.

The fact that the majority were burdened by the costs of care is probably primarily explained by the high proportion of individuals in our sample who were receiving immunomodulatory treatment. In addition, the costs of care are probably especially burdensome for the 42% in the sample who were not working.

The results derived from previous studies which reveal that PwMS exhibit high participation preferences [2,30] were confirmed by the fact that the great majority in the present study wanted to participate in planning their care. Involving PwMS in decision-making is included in the NICE-guidelines [28] and an increase in the proportion of people (63% in our sample) who actually participate in planning their care should be striven for.

During the study period there were no statistically significant variations in the proportion of PwMS with perceived needs with regard to various health-related services. However, over time, individual variations in perceived needs and in satisfaction with care were found. The reasons for these variations are not clear but might indicate that some needs are met or disappear while others vary as a consequence of the varying MS disease. All the same, variations in needs and satisfaction with care coincide with previous results concerning significant variations in functioning and disability in PwMS [31]. The results highlight the call for a flexible health care system that can provide services with a minimum delay and reduce the environmental barriers to optimized functioning.

In accordance with the NICE guidelines [28], a regular review of the individual's care needs should be offered. The majority perceived a need for rehabilitation, assistive devices, transportation service for the disabled, psychosocial support/counselling and information on social insurance/vocational rehabilitation at all points in time or sometimes, suggesting both that these are key services demanded by PwMS and also that evidence-based rehabilitation services e.g., exercise therapy [32], cognitive behavioural therapy [33] and multidisciplinary rehabilitation [34] should be provided more extensively in order to improve satisfaction with care.

PwMS who perceived a need at all times or sometimes, were most satisfied with the availability of assistive devices; workplace adaptation; home adaptation; and transportation service for the disabled, which indicates that the availability of these services may be considered as reasonably satisfactory within the Swedish health care system. However, these PwMS were least satisfied with the availability of psychosocial support/counselling; and information on social insurance/vocational rehabilitation which suggests a need for an increase in the supply of these services.

As can be expected, a severe state of MS was associated with a greater perceived need for almost all the health-related services studied; this result is in line with previous studies [9,14]. However, the fact that PwMS with a mild disease severity perceive a need for rehabilitation services to a lesser extent than those with a more severe state of MS might arise from expectations based on the way the health care system is structured to focus on symptomatic treatment rather than health promotion. The expectations and knowledge that PwMS have of the different health care providers' areas of expertise may have an impact on their perceived needs of different services as well as their satisfaction with care. Although a previous study revealed that a surprisingly large proportion of PwMS lack information about appropriate exercises [11], maintaining the level of functioning, with the assistance of e.g., a physiotherapist, may not be recognized as a need by PwMS with mild disease severity. PwMS risk a variety of secondary conditions, including physical deconditioning and weight problems [35], and a relationship between secondary conditions and health behaviour has been suggested [36,37]. Thus, as has been proved in a previous study [38], for people with disability a shift in focus from cure to care including health promotion, would presumably be effective in terms of outcome and costs.

The present study's major strengths were the longitudinal design and the low dropout rate in contrast to many previous studies in which postal questionnaires have been used [9,11,13,14]. Although the presence of a health professional may have influenced the responses, one advantage, considering that cognitive disability is common among PwMS, was the opportunity to explain the questionnaires. Furthermore, the professional's presence ensured that the questionnaires were filled in by the PwMS and not by proxies. In addition, the questionnaire included questions with different dimensions on both perceived needs and satisfaction with care. However, since our aim was to describe the perspective of the PwMS, we used a client-centred perspective and thus we do not know whether our results reflect the actual care received. Therefore, the present study should be complemented with a study of the use of care. Furthermore, the impact of met/un-met perceived needs and level of satisfaction with care on functioning and health-related quality of life should be explored.

The results are probably representative for a MS specialist outpatient clinic but there may be differences between countries or regions in Sweden due to different structures in the health care systems. The level of satisfaction with care in the present study may be influenced by higher demands made by PwMS attending a specialist clinic. On the other hand, at such a clinic the potential for meeting the needs of the PwMS ought to be most favourable and opportunities for improvements e.g., enhanced availability of rehabilitation services, are excellent.

## Conclusion

Despite the large proportions of individuals in our sample with mild disease severity, a considerable number of needs were identified of which many varied on an individual level over time. Rehabilitation, assistive devices, transportation service for the disabled, psychosocial support/counselling and information on social insurance/vocational rehabilitation, seem to be key services demanded by PwMS. Also the level of satisfaction with care varied and areas with a potential for improvement were identified e.g., availability of rehabilitation services including an increase in the supply of psychosocial support/counselling as well as information on social insurance/vocational rehabilitation. Future research is needed to explore how the different needs of PwMS best should be met in terms of outcome and cost-effectiveness and, within the different dimensions, what levels of satisfaction with care can be considered as acceptable.

## Competing interests

The authors declare that they have no competing interests.

## Authors' contributions

CY participated in the design and coordination of the study, performed the statistical analysis and drafted the manuscript. SJ participated in the design and coordination of the study, and helped to draft the manuscript. KG participated in the design of the study and helped to draft the manuscript. LWH participated in the design of the study and assisted in drafting the manuscript. LvK participated in the design and coordination of the study and helped to draft the manuscript. All authors read and approved the final manuscript.

## Pre-publication history

The pre-publication history for this paper can be accessed here:


